# Spatial and temporal dynamics of multidimensional well-being, livelihoods and ecosystem services in coastal Bangladesh

**DOI:** 10.1038/sdata.2016.94

**Published:** 2016-11-08

**Authors:** Helen Adams, W. Neil Adger, Sate Ahmad, Ali Ahmed, Dilruba Begum, Attila N. Lázár, Zoe Matthews, Mohammed Mofizur Rahman, Peter Kim Streatfield

**Affiliations:** 1 Geography, King’s College London, Strand Campus, London WC2R 2LS, UK; 2 Geography, College of Life and Environmental Sciences, University of Exeter, Rennes Drive, Exeter EX4 4RJ, UK; 3 Initiative for Climate Change and Health, International Centre for Diarrhoeal Disease Research Bangladesh, GPO Box 128, Dhaka 1000, Bangladesh; 4 Engineering and the Environment, University of Southampton, University Road, Southampton, Southampton SO17 1BJ, UK; 5 Social Statistics & Demography, University of Southampton, 58 Salisbury Rd, Southampton SO17 1BJ, UK

**Keywords:** Developing world, Environmental social sciences

## Abstract

Populations in resource dependent economies gain well-being from the natural environment, in highly spatially and temporally variable patterns. To collect information on this, we designed and implemented a 1586-household quantitative survey in the southwest coastal zone of Bangladesh. Data were collected on material, subjective and health dimensions of well-being in the context of natural resource use, particularly agriculture, aquaculture, mangroves and fisheries. The questionnaire included questions on factors that mediate poverty outcomes: mobility and remittances; loans and micro-credit; environmental perceptions; shocks; and women’s empowerment. The data are stratified by social-ecological system to take into account spatial dynamics and the survey was repeated with the same respondents three times within a year to incorporate seasonal dynamics. The dataset includes blood pressure measurements and height and weight of men, women and children. In addition, the household listing includes basic data on livelihoods and income for approximately 10,000 households. The dataset facilitates interdisciplinary research on spatial and temporal dynamics of well-being in the context of natural resource dependence in low income countries.

## Background & Summary

The well-being of populations living in geographically and socially marginalised regions across the globe are dominated by natural resource extraction, and use, and are heavily dependent on ecosystems and their services^1^. The relationship between productivity of natural resources and human well-being is complex, influenced by issues of access and ownership, and dynamic across time and space^2–8^. Hence there is a significant need to integrate analysis of ecosystem quality, the services ecosystems provide and the multiple dimensions of well-being that are concurrent with, or derived from, these ecosystems.

A diversity of studies to date have developed conceptual frameworks and collected data on specific elements of well-being and ecosystem services^9–13^. Much work on ecosystem services focuses on mapping ecosystem services and their joint production in bundles; on modelling the underlying ecosystem processes; and development of payment and market mechanisms^14,15^. The dataset reported here is one of the first large scale quantitative investigations that allows the nature and strength of relationships between ecosystem services and well-being to be tested, with generalisable results, and to include diverse indicators of well-being.

The dataset described in this paper was developed, designed and collected to represent multiple dimensions of well-being, ecosystem service use, social mechanisms, spatial variation and seasonality, in a context of social-ecological systems. The International Centre for Diarrhoeal Disease Research, Bangladesh (icddr,b), the University of Exeter and the University of Southampton co-designed and implemented the survey as part of the project *Assessing Health, Livelihoods, Ecosystem Services and Poverty Alleviation in Populous Deltas* (www.espadeltas.net) funded under the Ecosystem Services for Poverty Alleviation programme (www.espa.ac.uk) from 2012 to 2016. The project provides policy-relevant information on managing natural resources for well-being in Bangladesh, by developing systems models, scenarios and detailed analysis of poverty-ecosystem services linkages. The data reported have formed an integral part of these outputs and have been coupled with bio-physical models and scenarios of future climate and social change in an integrated systems model^16^ and directly analysed to determine the social and ecological conditions under which use of ecosystem services lead to positive well-being outcomes.

The quantitative household questionnaire survey was administered to 1586 households three times to capture temporal dynamics of well-being from ecosystem services. The sample was stratified by social-ecological systems in order to capture spatial dynamics of well-being from ecosystem services. Information on assets, income, expenditure, food consumption, satisfaction with life, blood pressure and anthropometric measurements of height and weight (in order to estimate the Body Mass Index) was collected to ensure representation of diverse forms of well-being. Detailed sections on fisheries and forest collection ensured that open access resources were fully represented. Multi-stage random sampling ensured that results were representative of the area and generalizable. Some data were collected about individuals to provide insights into intra-household dynamics.

The study area covered by the dataset is Khulna and Barisal Divisions on the southwest and south-central coast of Bangladesh, representing one of the most vulnerable parts of the Ganges-Meghna-Bhramaputra delta. Bangladesh scores low on international comparisons of human development and income, with low per capita income, although its average literacy and health outcomes (e.g. childhood mortality) score higher than expected for such low income levels^17^. The population of the non-urban delta region is dense with more than 800 people per square kilometre and with high incidence (>50%) of landlessness^18^. This area exemplifies many of the stresses associated with coastal and delta regions around the world: land use conflicts, large salinity fluxes with background increases in salinity, coastal hazards such as cyclones, prioritisation of monoculture to the detriment of open access resources, lack of government support and high population density^19^.

The diversity of variables, rarely collected together in the same survey instrument, create a high potential for reuse of this dataset. Furthermore, certain variables are comparable with the standard Household Income Expenditure Surveys of Bangladesh and the national census offering the possibility of analysing the data over multiple years or decades. The dataset can be used to test key associations between environmental processes and social and health outcomes that are critical for environmental policy and development strategies for Bangladesh, the delta region, and deltas more generally. For example, there are a range of variables that will allow researchers to examine the social relationships that affect livelihoods in rural areas such as money lending, casual labour, in-kind payments and share-cropping. Comprehensive data on yields, income and livelihood choices could be used to undertake simulation modelling of interventions, ranging from improvements in agronomy, through to social interventions in availability of credit, or supporting mobility and migration. The dataset here can be disaggregated by social-ecological system, and crucially includes information on seasonal variation in environmental conditions and livelihoods, a critical issue in the variation of well-being and poverty^20,21^.

## Methods

**Figure 1:**
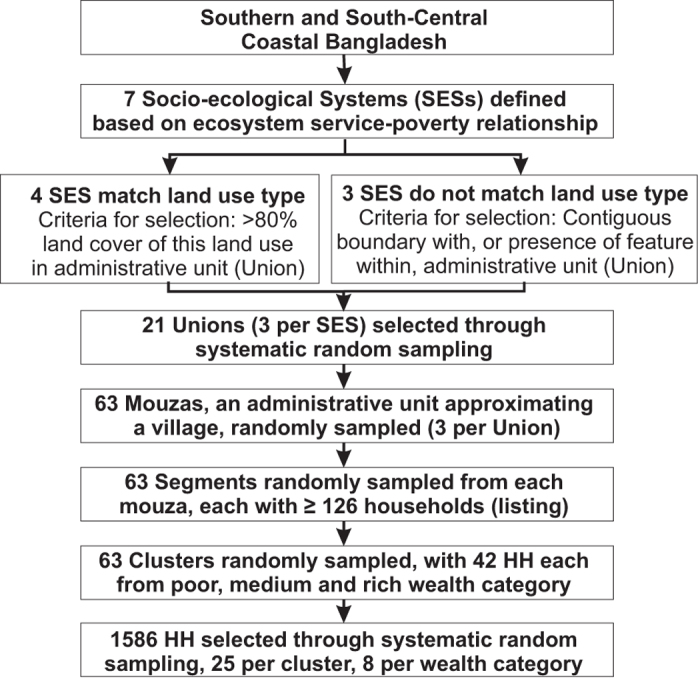
Schematic showing systematic sampling process. HH = households.

The following sections describe the sampling strategy used to randomly select households and individuals for the survey and survey implementation. Fig. 1 provides an overview of the sampling strategy.

### Defining social-ecological system strata

**Figure 2:**
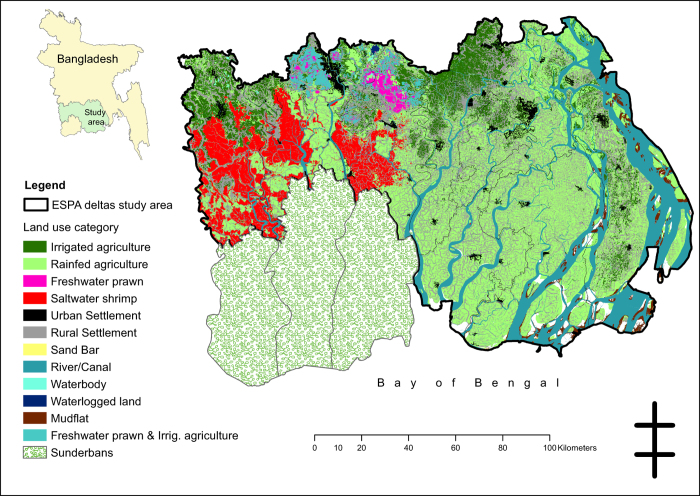
Land cover map of the study area used to define social-ecological systems.

**Figure 3:**
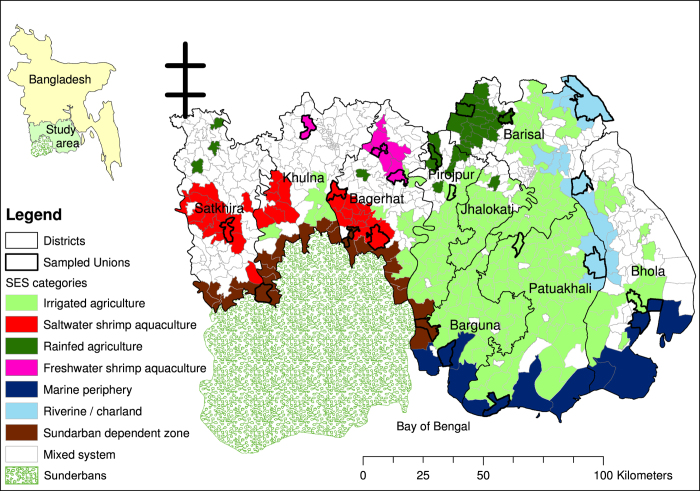
Unions in the study area assigned to social-ecological systems, with surveyed Unions highlighted in bold.

The first level of sampling was by social-ecological system. We assigned administrative units to social-ecological systems (SES) so that we could create a sampling frame. We did this by obtaining a land cover map (digitized from satellite imagery) of the field site and overlaying with a map of administrative units (Unions) in a geographic information system (ArcGIS) (see Figs 2 and 3). A Union is the smallest planning unit in Bangladesh, and covers several villages.

**Figure 4:**
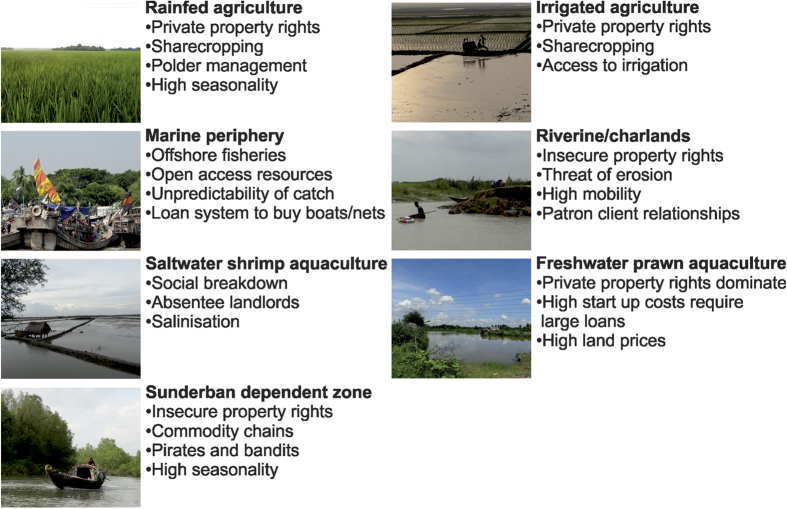
Brief characterisation of the social-ecological systems.

A social-ecological system is a set of ecosystems, human populations and institutions with close functional interactions^22^. Here, the concept is operationalized by integrating land cover with the social and livelihood systems that influence, and are influenced by, the way the land is used. The social-ecological systems of the field area were defined by a literature review and expert elicitation with scholars in Bangladesh, then verified through seventy-five semi-structured interviews carried out across the field site from September 2012 to April 2013 (Data citation 1). Seven systems were identified as having distinct ecological characteristics and access regimes: rainfed agriculture, irrigated agriculture, freshwater prawn, saltwater shrimp, riverine (including eroding islands), Sundarban mangrove dependence and offshore fisheries. Fig. 4 provides a brief summary of the social characteristics of ecosystem service use of each system.

Some social-ecological systems directly correspond to land use type, for example, shrimp aquaculture. However, in the case of coastal and offshore fishing, for example, fishermen live in villages among agricultural land and travel to the ocean to work. Therefore, two different methods were used in sequence to assign social-ecological systems to administrative units: land cover (agriculture or aquaculture) or proximity to land feature (Sundarban mangrove forest, coast of Bay of Bengal and riverine/charlands).

Each Union could belong to only one SES in this analysis, and the proximity to features was decided to be used as the more important factor if a Union could belong to multiple SES. If a Union did not have one dominant land cover type it was excluded from the analysis (white Unions in Fig. 3). ArcGIS was used to generate a Microsoft Excel file with the name of each Union and its assigned SES. The original land cover data by Union for the study area, and data processed for the sampling strategy can be downloaded from the ReShare data repository (espa_deltas_land_cover.xlsx, Data Citation 2). Table 1 shows the number of unions in each of the SES strata, and thus included in the sampling frame.

#### Social-ecological systems by land use

We generated an Excel file of land cover by overlaying the remotely sensed map of land cover onto Union boundary maps. Percentage land cover calculations were generated for the four SES that correspond to land cover, that is to say, aquaculture (freshwater and saltwater) and agriculture (irrigated and non-irrigated). Non-productive land cover uses were subtracted from total area (mudflats, rivers, canals, sand, rural and urban settlements, water bodies and water-logged land) before calculations took place. Urban areas were not included in the sample because of the nature of the research questions relating to direct natural resource dependence.

A sensitivity analysis was carried out to find out how many Unions fell within the sampling frame for each of the four SES if the percentage land cover threshold is changed. We investigated changes in the number of eligible Unions for 70, 80, 90 and 99 percent coverage of each of the four classes of land use. While 70 percent coverage may not allow the land use to be described as dominant, a threshold of 90 percent excluded too many Unions from the sampling frame. Thus, 80 percent coverage represents a balance and was applied in this research. The original land cover data is included in the Reshare database allowing other possible categorisations to be made (espa_deltas_land_cover.xlsx, Data Citation 2).

### Social-ecological systems by proximity to land feature

ArcGIS was used to manually select Unions adjacent to the Sundarbans, the southern coast or that contained a char (and thus create the riverine zone). Unions assigned to these categories were then excluded from the list of Unions belonging to land cover-SES. For example, once a Union was identified as a coastal Union, it was removed from the rainfed agriculture category. Or once a Union was identified as Sundarban dependent it was removed from the saltwater shrimp category.

Sundarban dependant zone included all the Unions adjacent to the Sundarbans directly or separated by the Baleshwar-Haringhata Rivers on the eastern edge of the forest. The Union on the south eastern corner of the forest that could be classified as either Sundarban dependent or coastal periphery, was categorized as a coastal Union based on expert knowledge. Unions on the Bay of Bengal from the western border of Bangladesh to Shahbazpur Channel were included in the sampling frame as marine periphery Unions. Four Unions were excluded from the sampling frame (in Char Manpura Upazilla and Char Khukrimukri) because of known security issues.

We used a pre-existing database of chars^23^ to classify riverine char Unions. A char is a low lying area within a river that may be seasonally flooded, be attached to the riverbank or exist as an island. The chars were mapped as point features in a GIS and classified as attached to mainland, riverine, and marine or estuarine. We were interested only in the dynamics of riverine chars so we excluded the marine and estuarine chars from the analysis. The presence of chars was verified using an up-to-date land cover map.

The list of social-ecological systems is not exhaustive. The systems chosen represent a compromise between the sample size (and thus possible number of strata), the ability to define the systems in a GIS (to create a robust sampling frame), and systems that were hypothesized to influence the poverty-ecosystem service dependence relationship. For example, while inland capture fisheries are an important system, they are ubiquitous across the study area and so were not included as a separate category. Wetlands (*beels*) are also present in the northern part of the field area, however, their geographical spread was small, their importance within the region limited, and there was no way of isolating them in the GIS for systematic random sampling so they were omitted. Some land cover types such as waterlogging, were not associated with separate hypotheses regarding the relationship between ecosystem services and well-being based on the initial qualitative work.

### Systematic random sampling

Once the super strata were generated, households to interview were identified through random cluster sampling by Union, then Mouza (approximating a village) and clusters. The total sample size was spread evenly between social-ecological systems to ensure that all systems have equal representation in the analysis despite uneven geographical extent. Therefore, certain social-ecological systems are over-sampled and others under-sampled.

The sample size required was calculated using prevalence of poverty according to a Head Count Ratio. Prevalence of poverty according to Head Count Ratio below lower poverty line (constructed on the basis of cost of basic food needed) is 26.7 percent and 15.4 percent respectively^24^ for Barisal and Khulna Division. Here nine districts (3 from Khulna Division and 6 districts from Barisal Division) were considered as one study area and the mean was weighted accordingly. Weighted mean of prevalence of poverty below the lower poverty line is 22.1 percent for the project area. A further 10 percent was added to the sample size to take into account non-responses, 6.5 percent for precision and additional 10 percent more was added to maintain sample size between the different rounds of the survey once migration was taken into account. The formula used to determine the sample size is:

$$n=p(1-p){\left(\frac{Z}{d}\right)}^{2}$$

Where,

p=estimated proportion (from a survey);

z=z value associated with the degree of confidence selected;

d=allowable error

Unions belonging to each of the seven SES were labelled from 1 to n (west to east). The.dbf files from the GIS were converted to Microsoft Excel files and filter was used to arrange the Unions from one to n by SES. Table 2 shows the number of Unions in each of the SES. Three Unions in each SES were systematically selected. The first Union was selected randomly within the range 1 to k using R programme, and then every kth Union was selected, where k=No. of Unions within the SES/3. The number of Unions per SES was limited to three because of time and cost constraints. If any Union selected had less than three Mouzas with 150 households (the segment size) then we excluded that Union and considered the nearest Union based on the criteria mentioned above.

To select Mouzas from each Union, first any Mouza with fewer than 150 households identified using the Mouza list from the Bangladesh Population Census 2011 Tables^25^, was removed. Then the remaining Mouzas were assigned number from 1 to n. R programme was used to randomly selected three Mouzas from each of the selected three Unions. Each Mouza was divided into segments, where number of segments=total no. of households in the selected Mouza/150. Due to time and money constraints a segment was considered, not an entire village. A segment of 150 households was decided based on previous studies in Bangladesh^26^. R programme was used to randomly select a segment. The count of the households began from the north corner of the Mouza and moved southwards. Full details of the villages in which the survey took place, can be found in the ReShare repository (espa_deltas_locations.xlsx, Data Citation 2).

Thus 63 segments, randomly selected from the 63 Mouzas, were mapped and then listed by trained enumerators. The listing form included information on name of main earner and household head, age, marital status, primary, secondary and tertiary occupation of main earner, monthly total household income, and household construction material information. The full household listing data is available from the ReShare repository (espa_deltas_listing.csv; espa_deltas_listing.sav, Data Citation 2). The household listing facilitated systematic random sampling of households. No further stratification took place.

Households were selected where there was the presence of both a male aged 18 to 54 and a female respondent aged 15–49. The target respondent for the survey was the main earner, not necessarily the household head, although the two categories often overlap. The main earner (male or female) completed the structured questionnaire. Information on global satisfaction of life, anthropometry (height and weight) and blood pressure was collected from both a male and female member of the selected household.

If the main earner was not available at the time of interview then the enumerator made an additional two attempts to catch him or her at home. However, if the main earner was not likely to return to the household during the time enumerator was in the area, then the spouse of the main earner, second earner or spouse of the second earner of that household was interviewed (in that order of preference), as long as he or she was between the ages of 18–54 years for men and 15–49 for women. If no-one fitting this description was in the household we excluded that household and moved to the next selected household.

### Ethical approval

Prior to implementation the research protocol was subject to review and approval by the Research Review Committee (RRC) and the Ethical Review Committee (ERC) at icddr,b. In addition, in order for the research to be approved by the ERC, all named researchers on the research protocol completed National Institutes of Health online training and individual ethical approval was obtained from the University of Southampton (as the lead institution in the ESPA Deltas consortium in the UK) and the Ecosystem Services for Poverty Alleviation Directorate.

The ERC, an independent body to safeguard the physical, mental and social well-being of the participants, is guided by the relevant international regulations and is responsible to the Board of Trustees of icddr,b. The committee reviews each protocol involving human participants and accords approval, and the decision of the ERC in this matter is final. The icddr,b ERC is internationally recognized ethics review committee and pioneer in Bangladesh. The icddr,b ERC is a registered Institutional Review Board with Federal Wide Assurance (#FWA0001468), Human Welfare Assurance (#IRB00001822) since November 2001.

### Survey implementation

The ESPA Deltas social survey was implemented in the south west and south central coastal zone of Bangladesh and included rural areas of Satkhira, Khulna and Bagerhat districts of Khulna Division and all districts in Barisal Division except Jhalokati. The survey was implemented with the assistance of Associates for Community and Population Research (ACPR), a highly experienced data collection firm in Bangladesh.

ACPR together with icddr,b provided intensive training on survey tools, data collection methodology and ethical grounds of social data collection. Several days of field testing of the survey tools were carried out before each round of survey as there were minor modifications of questionnaire in each round of survey (e.g. the gender empowerment and response to oil spill sections were added in third round of survey). A field guide was distributed to teams carrying out water sample collection and measuring blood pressure in Bengali and English. The questionnaire was pretested in the field in a pilot phase, before data collection.

Thirty skilled field staff were recruited along with ten experienced supervisors for the study and retrained on standard methods of obtaining physical measurements. Seven teams were assigned to seven zones, each team consisted of one supervisor, three interviewers and one porter to carry height scale and other equipment. To ensure the quality of the data, a monitoring team from icddr,b checked one percent of the data and held periodic meetings to provide necessary feedback to the field work. Prior to data collection from individual household members, written consent was taken in presence of a witness. In the second and third round surveys enumerators tried to reach the same household and interview the same household members as in the first round. Three to five percent of households were absent in the second and third round for reasons such as permanent or temporary migration, or unwillingness to give the interview.

All study participants were interviewed only after giving informed consent according the Belmont principles of respect for persons and using consent forms approved by the Ethical Review Committee of icddr,b. In addition, where applicable, assent as also taken. Efforts were made to ensure that all respondents were appropriately informed about the study and thoroughly understood their participation in the study. Participation was voluntary and interviewers ensured that participants knew that refusal to participate would not lead to any adverse consequences. According to ERC requirement, one copy of the signed informed consent form was handed over to all potential study participants.

Height and weight were collected from respondent and his or her counterpart. These measurements were also taken from eldest under five children if available. If the eldest child under five was less than a year old, then length was measured instead. While measuring height, the Frankfort position was confirmed and reading was noted accurately. Standard tools were used to measure for height (stadiometer) and weight (Uni Scale by Seca).

The three rounds of survey were implemented between June 2014 and March 2015 (see Table 2). icddr,b closely monitored the entire survey. Direct observation or spot checking in selected villages, and re-interviewing with a quality control questionnaire in selected households, formed part of monitoring process. Survey data and accompanying questionnaires are available on the ReShare Depository (espa_deltas_all_data_1st_round, espa_deltas_all_data_2nd_round, espa_deltas_all_data_3rd_round (.sav &.csv files), Data Citation 2).

### Code availability

This study did not use any computer codes to generate the dataset. The IBM SPSS Statistics (version 22) software was used to store and quality check the collected data.

## Data Records

Three different datasets are available in association with this research. The first contains the data required to sample by social-ecological system (ESPA_DELTAS_LAND_COVER.xlsx, Data Citation 2). The second data record contains the results of the household listing (espa_deltas_listing.csv; espa_deltas_listing.sav Data Citation 2). While it provides only limited data, the large sample size may facilitate analysis. The final data records provide the survey results (espa_deltas_all_data_1st_round, espa_deltas_all_data_2nd_round, espa_deltas_all_data_3rd_round (.sav &.csv files), Data Citation 2). The Survey data is provided in IBM SPSS and comma-separated values format. All 3086 variables are named according to their number in the questionnaire, and fully described in the variable labels. The household listing and three survey instruments can be downloaded in English and act as the code book for the datasets.

## Technical Validation

The quality of the dataset is ensured by: a) thorough pre-testing of the questionnaire; b) translating the questionnaire into Bengali, including local terminology, and reverse translating to check quality of translations; c) recruitment of experienced enumerators and comprehensive training in survey implementation; d) quality control questionnaires being carried out alongside the main data collection and high levels of supervision in the field; e) double data entry and numerous quality checks on the final digital dataset, including cross-referencing original paper surveys. These are detailed in the following paragraphs.

The survey questions were designed based on the research questions of the project, using questions from other surveys already implemented in Bangladesh where appropriate, and drawing on the qualitative data collection and expert judgement to create new questions. To ensure that the questions are relevant and meaningful, extensive pre-testing of the quantitative questionnaire was conducted in the study area prior to finalisation of the survey questions.

Training of the enumerators is essential for effective implementation of a survey. A deep understanding of the questions and philosophy of the survey ensures that enumerators are able to help the surveyed households in answering the questions properly. To achieve this, the enumerator team was selected for its long track record in doing similar surveys and was trained over a period of a month by the ESPA Deltas research team. Role play and field practice was carried out for every section of the questionnaire. Specialists were brought in as required, for example doctors for the blood pressure measurement and experts in using global positioning systems for the location data. Rigorous training on anthropometry was carried out.

A quality control team was assigned in the field to monitor data collection. Spot checking, direct observation and re-interviewing of five percent were carried out. A senior level supervision team also frequently visited the data collection activities in the field. During the survey the field supervisor checked all completed questionnaires. The interviewer cross-checked each questionnaire for internal consistency at end of the day. Section 3 of the survey provides general information on the range of incomes which subsequent sections investigate in more depth. As such, Section 3 was used (with other key indicators) to check that all appropriate income and expenditure questions had been completed. If not, the respondent returned to the household. Analysis of these variables reveals a highly detailed data set that has captured household differences For example, attributes such as monthly income and food expenditure show high variance between households despite a four month recall period.

Completed questionnaires were checked before data entry by an office editor. In the process of data entry all possible logical checks were built into the program (CSPro). Dual entry and comparison between datasets ensured correct data entry. Data management experts from icddr,b thoroughly checked all possible inconsistencies (e.g. range checks, conditional checks such as checking that only females have given birth, identification of outliers) of the datasets in an ORACLE platform. The survey used paper for recording the data. The original paper version is kept to allow the team to check individual records in the digital dataset if necessary.

Finally, the dataset was thoroughly analysed for outlier and extreme values to ensure that typing errors are eliminated from the final dataset. To identify such typing errors, individual and composite variables were calculated and summarised as minimum, median, mean, maximum and compared with other data sources and reports or, if similar data was not available for Bangladesh or the study area, these were evaluated by expert judgement.

## Usage Notes

### Data access conditions

A benefit of the data is their spatial nature that allows social factors to be analysed in the context of environmental conditions and resources. Therefore, the location of the villages is included in the dataset. However, this increases the sensitivity of the data as it creates the potential for households within each village to be identified from the survey data. As such, the data has been made available as safeguarded on the UK Data Archive’s data repository ReShare. In order to download safeguarded data the user must register with the UKDA and agree to the conditions of their End User Licence (For conditions of the End User Licence see: https://www.ukdataservice.ac.uk/get-data/how-to-access/conditions#/tab-end-user-licence). For commercial use, please contact the UK Data Service at help@ukdataservice.ac.uk.

### Potential for double counting

The survey focused on collecting in-depth information on the ways people use natural resources. A system of skips and checks were put in place to improve the survey experience for the respondent which creates the potential for double-counting when analysing ecosystem services based income data. Section 3 of the survey collects information on income of all household livelihood sources. If the respondent mentions agriculture, aquaculture, fishing or mangrove forest collection activities in Section 3, then the corresponding part of Section 4 is also completed. Therefore, data on income from agriculture, aquaculture, fishing or mangrove forest collection is collected twice: first as a rough estimate in Section 3, and then in more depth in Section 4. Thus, either the information from Section 3 or from Section 4 can be used (but not both).

### Differences between rounds

Due to attrition of households between survey rounds, not all cases are present in all three rounds. Data should be filtered before proceeding before carrying out longitudinal analysis on any of the variables. An additional 10 percent was added to the initial sample in order to account for expected attrition. Actual attrition rates were much lower: 4.4 percent between the first and second rounds, and 3.5 percent between the first and third rounds.

There are three rounds of data for all sections except a section added specifically to capture the impact of an oil spill that occurred in the Sundarban mangrove forest between the second and third rounds; and the gender empowerment section that was only carried out once, in the final round. There were some additional data collected in round three to try and better capture variables of interest that were not well captured in previous rounds. Whereas in Round 1 and 2 information was only captured on economic migrants (i.e. migrants who were remitting to the household), in Round 3 households were asked to list all family members who had left the household and were now living away.

Since the number of people using the mangrove forest seemed improbably low in the first two rounds, additional prompts were added in the final round. In Round 1 and 2, questions regarding Sundarban forest collection (in Section 4) were asked only to those who had mentioned that they use the forest in the previous section (as with all other ecosystem-based incomes). However, in Round 3 all households in the Sundarban dependent zone Unions were asked the questions in Section 4, regardless of whether they had mentioned using forest resources in the previous section.

## Additional Information

**How to cite this article**: Adams, H. *et al.* Spatial and temporal dynamics of well-being, livelihoods and ecosystem services in coastal Bangladesh. *Sci. Data* 3:160094 doi: 10.1038/sdata.2016.94 (2016).

**Publisher’s note**: Springer Nature remains neutral with regard to jurisdictional claims in published maps and institutional affiliations.

## Supplementary Material



## Figures and Tables

**Table 1:** Number of Unions in each social-ecological system.

**Social-ecological system**	**Number of Unions**
Coastal periphery	11
Sunderban dependent zone	24
Riverine	17
Rain-fed agriculture	223
Freshwater prawn	11
Irrigated agriculture	29
Saltwater shrimp	31

**Table 2:** Timing, recall period and surveyed population in each survey round.

**Rounds of survey**	**Period of implementation**	**Months captured in recall questions**	**Surveyed population**
First round	Jun-14	February –June	1586
Second round	Oct-Nov 2014	June -October	1516
Third round	Mar-15	October-February	1531
